# Supplementary material for: napariTFM: An open-source tool for traction force microscopy and monolayer stress microscopy
Source: PLoS Comput Biol. 2026 Mar 16;22(3):e1014045. doi: 10.1371/journal.pcbi.1014045 (PMC13020755; doi:10.1371/journal.pcbi.1014045)
Supplement: Attachment — Submitted filename: napariTFM_response_to_reviewers.pdf [file pcbi.1014045.s001.pdf]

Reviewer #1: The authors present a user-friendly graphical interface for estimating cellular traction forces from TFM images and describe its validation. The motivation is sound, as existing traction force algorithms are technically demanding and often inaccessible to experimental biologists. The proposed tool may indeed lower this barrier and contribute to wider adoption of TFM and MSM analyses.

However, the manuscript does not meet the PLOS Computational Biology criteria of “providing new biological insights” or “bringing exceptional new capabilities.” The presented work primarily integrates existing algorithms within a GUI rather than introducing novel computational approaches or enabling new biological discoveries.

We thank the reviewer for recognizing the value of napariTFM in lowering barriers to TFM and MSM analysis for experimental biologists. We agree that napariTFM integrates existing algorithms rather than introducing novel computational methods. We believe this contribution is well-suited for the Software section of PLOS Computational Biology, which both the editor and Reviewer 2 have suggested as an alternative placement for this manuscript. The manuscript should have been submitted there from the start and we apologize for this oversight.

The Methods section includes detailed mathematical formulations, but all algorithms are established methods from previous studies. As none are original, this section could be condensed with appropriate citations instead of full equation listings, which currently risk confusing readers.

We agree with this assessment and have revised the Methods section accordingly.

Concretely, we have removed technical implementation details from “2.4 Traction Force Reconstruction” and from “2.5 Monolayer Stress Microscopy Implementation” (now just “Monolayer Stress Microscopy”)

In Figure 3, the validation using three displacement scenarios (low, medium, high) lacks justification, and the quantitative differences between these conditions are unclear. The use of correlation coefficients as the sole evaluation metric is problematic, as it measures only qualitative similarity. Quantitative error measures such as MSE or RMSE should be provided, and the method of calculating strain energy should be clarified.

The three displacement scenarios were selected to span the practical measurement range encountered in TFM experiments and to evaluate algorithm performance across distinct regimes. We revised all scenarios to cover three orders of magnitude in applied force (0.25 μN, 0.025 μN, and 0.0025 μN) applied on a 3 μm radius circle. The low displacement scenario was reduced to approach the lower sensitivity limit of the TV-L1 optical flow algorithm, representing challenging sub-pixel displacements. The high displacement scenario was increased to better probe the upper limit of reliable tracking, where large deformations can introduce significant errors. The medium scenario was adjusted slightly to

lie logarithmically between the two, representing the optimal operating regime. These changes are now clearly described in the Methods section, with quantitative displacement magnitudes reported for each condition.

We agree that correlation coefficients alone are insufficient for comprehensive validation. RMSE would require normalization (NRMSE) to compare different conditions, but even then it does not accurately reflect measurement performance because displacements vary widely across the field, from near-zero regions to regions of high displacement. Normalizing by the displacement in low-magnitude regions artificially inflates the error, and separating the map into regions is difficult due to smooth transitions. Instead, we report error as a function of ground-truth displacement magnitude. For forces, the situation is different: forces are mostly constant in adhesion regions and zero elsewhere. Here, we divide the map into adhesion and background regions and quantify error using the metrics defined by Sabass et al. (Biophysical Journal, 2008). In the revised manuscript, we now include these quantitative error metrics (DTM, DTPS, and DTA) for both displacement and force maps.

We also removed the strain energy calculations. These were supposed to serve as quantitative error assessment and are now no longer necessary.

Concretely, we added the technical details about the synthetic data creation to paragraph 2.6 "Synthetic Data Generation for Validation", revised figure 3 to include these new error metrics, added a new paragraph to section 3.1 "Displacement Analysis Performance" and revised section 3.2 "Traction Force Reconstruction Accuracy to describe these.

Figure 4A shows nearly uniform stress distributions, raising doubts about the relevance of the test case. Similar concerns apply to the reliance on correlation coefficients in Figure 4. While the moving-cell model in Figures 4C–D is a positive addition, it still demonstrates software validity rather than "exceptional capability".

The nearly uniform stress distribution of the square plate problem was chosen because it has a known analytical solution. This is the same benchmark used to validate the original MSM algorithm in pyTFM.

That said, we had the same concerns about the relevance of this scenario, as it is quite removed from realistic cell conditions. For this reason, we collaborated with Dennis Wörthmüller to generate finite element simulations of cells on micropatterned substrates (Figures 4C–D), which provide more realistic stress distributions. These simulations have the advantage of being biologically relevant, though the ground truth is numerical rather than analytical.

Together, the two approaches complement each other: one provides exact validation, the other provides realistic validation. We note that the original pyTFM publication did not include this additional realistic validation.

To address the reviewer's request for additional quantitative validation, we used metrics consistent with those previously applied in the displacement analysis. For the square plate benchmark, we report mean absolute and mean relative error to quantify accuracy in the

uniform stress field. For the FEM simulations, we compute error as a function of ground-truth stress magnitude by binning stress values and calculating the mean error within each bin.

This approach provides a clearer picture of stress recovery performance. It reveals that regions of low stress exhibit high relative error due to low normalization denominators, while error decreases at higher magnitudes and converges to approximately 25% stress underestimation for substantial stress values.

Concretely, we revised figure 4 to include these new error metrics and added a new paragraph to section 3.3 “Monolayer Stress Microscopy Performance” to describe these.

Figure 5 is described as showing spatial propagation of contractile forces, but the figure and description do not clearly support this claim.

We agree with the reviewer that the original Figure 5 did not clearly support the claims made. We have replaced this figure entirely with a reanalysis of the epithelial doublet data from figure 1 of Ruppel et al. (eLife 2023), allowing direct comparison between napariTFM results and the published findings.

Concretely, we changed figure 5 and wrote a new corresponding results section 3.5 “Validation Against Published Experimental Data”

As a minor issue, the reference list does not follow journal format: only the first six authors should be listed, followed by “et al.” Reference 22 and several others should be corrected accordingly.

We thank the reviewer for pointing this out and apologize for the oversight. We corrected the relevant references.

In summary, while the software may have practical value, the manuscript lacks computational or biological innovation required for publication.

We thank the reviewer for their thorough evaluation. We acknowledge that napariTFM does not introduce novel algorithms, and we agree that the Software section of PLOS Computational Biology is a more appropriate venue for this work.

However, we respectfully emphasize that the contribution lies not only in practical accessibility but also in technical improvements over existing tools, particularly for MSM analysis. For MSM analysis, the only freely available alternatives are pyTFM (Bauer et al., PLOS Computational Biology 2021) and iTACS (Nguyen et al., Jove 2022). iTACS integrates with Micro-Manager, making it less accessible as a standalone analysis tool. Compared to pyTFM, napariTFM improves upon the implementation by using gmsh for mesh generation, which provides better mesh quality and user control over mesh parameters, and by leveraging numba for just-in-time compilation, resulting in significantly faster computation times. This makes it much more suitable for analysis of large datasets, especially important for time-series data.

Furthermore, we provide a level of systematic validation that exceeds what is typically provided for TFM and MSM software. It has historically been the norm in the field to use TFM and MSM tools that are poorly validated against ground truth data. We believe the systematic validation presented here goes beyond what is typically provided for such tools.

Beyond confirming software accuracy, our validation characterizes the measurement limits of the implemented algorithms across a displacement range spanning approximately 0.2 to 30 pixels. The error analysis reveals an optimal operating regime of roughly 1–10 pixels of displacement, where measurement errors are minimized. These findings provide practical guidance for experimental design: researchers can use this information to select substrate stiffness such that expected cellular forces produce displacements within the optimal measurement range.

We hope the revised manuscript, with additional quantitative metrics, improved high-displacement validation, and new experimental validation against our previously published data in Figure 5, addresses the reviewer's concerns.

Reviewer #2: Traction force microscopy (TFM) and monolayer stress microscopy (MSM), which builds on it, have become standard tools in the field of mechanobiology, but as the authors correctly point out, what is missing is an accessible and GUI-based tool to run such reconstructions without having to worry much about the underlying methods and limitations. The new software napariTFM presented here fills this gap and could become a very useful resource for a large user community. The software puts together the most advanced solutions in this field and is available on GitHub. I did not have any trouble to install it. In the manuscript, the authors verify their software with simulated data and using experimental data from their own recent paper in eLife (Ruppel, Artur, et al. "Force propagation between epithelial cells depends on active coupling and mechano-structural polarization." Elife 12 (2023): e83588). In my assessment, this could be a very useful methods paper for a broad user community.

We thank the reviewer for their positive assessment and for taking the time to install and test napariTFM.

I have a few comments that should be addressed in a moderate revision.

First I note that the authors provide the software, but not the image data. All data files described in the manuscript should be made available in the GitHub repository and included in the ZIP-file. I particular, I note that Fig. 1 gives specific file names like beads.tif, so it would be nice to be able to try the software out with such files.

We thank the reviewer for pointing this out. The example data is currently included in the repository under _validation/doublet_example/, but we acknowledge this is not clearly documented or easily discoverable. Furthermore, we noticed that some of the files have failed to upload. In the revised manuscript, we now host all example datasets on Zenodo (https://zenodo.org/records/18390989), with a dedicated DOI and provide explicit links in

both the manuscript and the GitHub README. This includes the files referenced in Figure 1 (e.g., beads.tif) so that users can immediately test the software with real data.

Regarding the image processing part, I wonder if it would be possible to add other methods than the optical flow routine. For example, in TFM, often one also uses Digital Volume Correlation (compare e.g. the work of Christian Franck). Naparia seems to be a good environment to provide other options for image processing; maybe some of these are already available through other plugins.

We agree that napari provides an excellent environment for integrating alternative displacement detection methods. While Python implementations of DIC exist (e.g., muDIC, spam), the ecosystem is less mature than for optical flow. For the current version, napariTFM uses the TVL1 optical flow algorithm from OpenCV, which is implemented in C++ and thus offers significant performance benefits. The modular structure of napariTFM is designed to accommodate alternative methods, and we mention this as a direction for future development in the revised manuscript.

We have added a paragraph discussing this to the Discussion section

In the introduction, the authors should also mention the software provided by Benedikt Sabass on GitHub, https://github.com/CellMicroMechanics, especially for BayesianTFM (although it requires the commerical software Matlab, this code seems to be used widely).

Also in the introduction, BEM should be cited before FTTC and with Dembo BPJ 1999, because this was the very beginning of TFM. For FTTC, one should also cite Sabass BPJ 2008. For FEM, a pioneering paper was Yang, Z., Lin, J. S., Chen, J., & Wang, J. H. (2006). Determining substrate displacement and cell traction fields—a new approach. Journal of theoretical biology, 242(3), 607-616. In general, the manuscript would benefit from more references throughout.

We thank the reviewer for these suggestions. We have added a reference to the Bayesian TFM software by Benedikt Sabass in the introduction. We have also revised the order of citations to reflect the historical development of TFM methods, now citing BEM first with Dembo et al. (1999), followed by FTTC with Sabass et al. (2008), and including Yang et al. (2006) for FEM-based approaches. We have added additional references throughout the manuscript as suggested.

References are not always complete. For example:
Ref. 15: book title missing
Ref. 22: journal title etc missing

We have corrected the incomplete references, and apologize for the oversight.

The concept and need for regularization should be explained before methods for it are introduced.

We agree and **we have revised section 2.4 Traction Force Reconstruction to introduce the need for regularization.**

I am a bit unsure if "research paper" is the right category for this work. PLOS CB also has the categories "Methods" and "Software". Maybe the latter would fit better.

We agree with the reviewer and the editor that the Software section is a more appropriate venue for this work. The manuscript should have been submitted there from the start and we apologize for this oversight.
